# Pulmonary artery involvement in Behçet's disease: A challenging case and comprehensive management approach

**DOI:** 10.1016/j.radcr.2024.02.054

**Published:** 2024-03-08

**Authors:** Kayla A Aikins, Zoé N. Anderson, Benjamin L Bosse, Susan Knowles

**Affiliations:** a University of Nevada, Reno School of Medicine, 1664 N Virginia Street Reno, NV 89557; b Carson Tahoe Rheumatology, 2874 N Carson St Ste 200, Carson City, NV 89706

**Keywords:** Behçet's disease, Systemic inflammatory vasculitis, Pulmonary artery involvement, Autoimmune process, diagnostic radiology, Aphthous ulcers

## Abstract

A 26-year-old male with Behçet's disease (BD) presented with recurrent oral and genital ulcers, bilateral pneumonia, and a left lower pulmonary artery aneurysm. Endovascular coil embolization was initially performed, followed by treatment with prednisone, colchicine, and azathioprine. Despite treatment, disease progression occurred, requiring additional embolization, intravenous pulse methylprednisolone, and cyclophosphamide. Ultimately, a combination of medical and endovascular interventions resolved the pulmonary arterial aneurysms. This case highlights BD's systemic inflammatory nature and vascular complications like pulmonary artery aneurysms. It emphasizes the importance of early detection and individualized, multidisciplinary care for such complications.

## Background

Behçet's disease is a rare systemic inflammatory vasculitis with diverse clinical manifestations, including potential involvement of the pulmonary arteries [1]. The etiology of Behçet's disease remains uncertain, but it is believed to arise from an autoimmune process triggered by infectious or environmental factors in genetically susceptible individuals [2]. The disease can affect both arteries and veins, leading to various symptoms, such as aphthous ulcers, genital ulcers, ocular lesions, deep venous thrombosis (DVT), and pulmonary artery involvement (PAI). Management of Behçet's disease is complex and must be tailored to individual patients, considering organ system involvement, gender, and relapsing and remitting symptoms. PAI poses significant challenges and is of interest due to its elevated mortality risk [3]. It is a relatively rare complication, observed in less than 5% of Behçet's cohorts [4,5]. This case offers valuable insights into the challenges of managing pulmonary artery manifestations in Behçet's disease and emphasizes the significance of a comprehensive and individualized approach to patient care.

## Case report

**Fig. 1:**
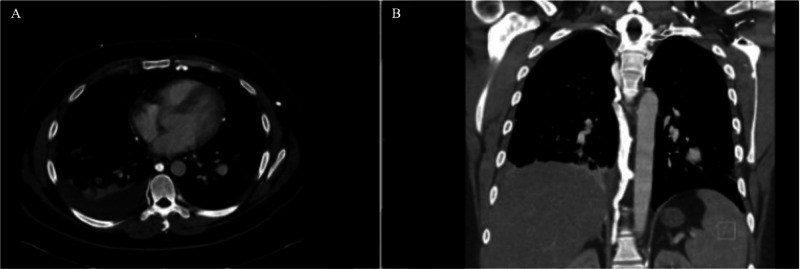
CTA Chest with pulmonary artery imaging with intravenous contrast. Axial (panel A) and coronal (panel B). A pulmonary artery aneurysm measuring 1.6 cm was identified in the left lower lobe. There is evidence of chronic, complete occlusion of the SVC, accompanied by the presence of extensive collateral veins observed in the chest wall, mediastinum, pericardium, and upper abdomen.

A 26-year-old male presented to his primary care physician with recurrent oral and genital ulcerations, along with symptoms such as fatigue, malaise, and weight loss. Elevated inflammatory markers led to a referral to rheumatology. During the wait for rheumatology evaluation, the patient developed respiratory symptoms, including a cough, hemoptysis, chest pain, and fever. On hospital presentation, he appeared chronically ill, displaying low-grade fevers below 100.5, daily sputum production, right upper chest wall pleuritic pain, and fatigue. CT angiography (CTA) revealed bilateral pneumonia, pleural effusion, and incidentally discovered a 1.6-1.9 cm left lower pulmonary artery aneurysm, with superior vena cava (SVC) occlusion showing collateralization (Fig. 1).

Upon evaluation, the patient was identified as a candidate for endovascular intervention for the incidental pulmonary aneurysm, raising suspicion of undiagnosed Behçet's disease. Considering limited data on treatment based on aneurysm size and recognizing the life-threatening potential for rupture, it was decided to proceed with aneurysm intervention following pneumonia resolution with antibiotics. Two months later, endovascular coil embolization of the aneurysm was performed.

**Fig. 2:**
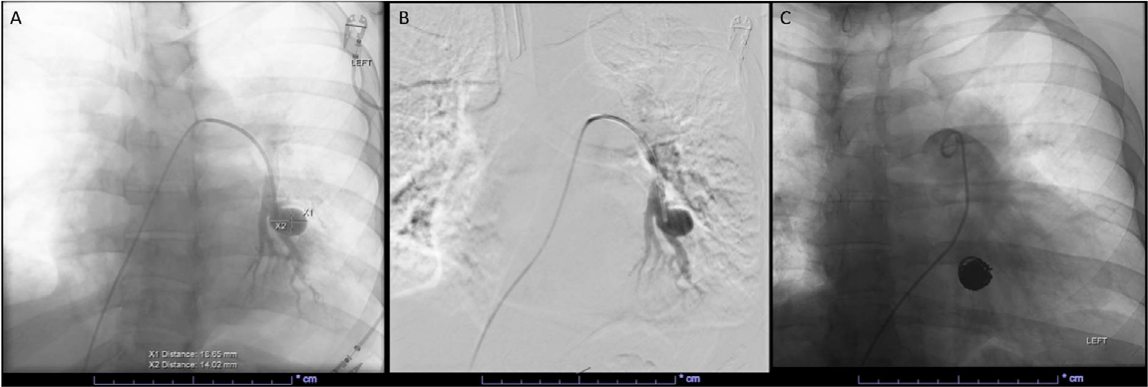
Three images collected from a fluoroscopically guided pulmonary artery aneurysm coil embolization. Panel A-B: Catheter placed in the left pulmonary artery proximal to the left lower lobe anterobasal and lateral segmental branches. Contrast injected into the vessel to visualize and measure the aneurysm (18.65 x 14.02 mm). Panel C: catheter placed in the proximal left pulmonary artery hand injecting contrast visualizing the placement of multiple embolization coils.

Subsequently, a diagnosis of Behçet's disease was established based on clinical presentation and positive HLA-B51. Initial treatment with prednisone, colchicine, and azathioprine aimed to suppress the disease and reduce aneurysm size, preventing progression or recurrence. However, follow-up CTA revealed disease progression with the development of new pulmonary artery aneurysms. Additional endovascular coil embolization for multiple aneurysms was performed later that year (Fig. 2).

**Fig. 3:**
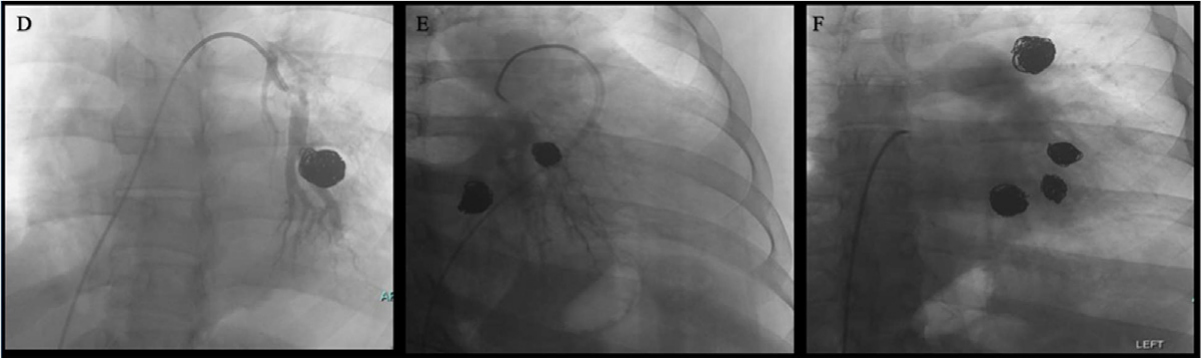
Three images collected after a fluoroscopically guided pulmonary artery aneurysm coil embolization. Panel D-F: embolization coils placed in left pulmonary artery in left lower lobe anterobabsal and lateral segmental branches without appearance of new aneurysms or aneurysm flow.

To address refractory disease, the patient received intravenous pulse methylprednisolone and a 6-month course of intravenous cyclophosphamide. Follow-up CTA showed resolution of previously coiled pulmonary artery aneurysms and the absence of new aneurysms (Fig. 3). This outcome underscores the necessity and efficacy of a combined approach involving both coil embolization and medical management. The patient was successfully transitioned to adalimumab and azathioprine, enabling him to taper completely off prednisone.

## Discussion

Behçet's disease, a systemic inflammatory disorder, presents significant vascular implications, notably the potential development of pulmonary artery aneurysms. On CT, a pulmonary artery aneurysm is identified by focal dilation beyond 29 mm in diameter in adults and 17 mm in the interlobar pulmonary artery [6]. In Behçet's disease, aneurysms typically manifest in the right lower lobe, marked by recurrent thrombosis, and inflammation. The involvement of transmural necrosis and obliteration of the endarteritis of the vasa vasorum is believed to play a critical role, compromising the vessel wall's integrity. These aneurysms, though rare, pose a potentially life-threatening risk, characterized by saccular outpouching of the pulmonary artery, indicative of a contained rupture. The mortality rate associated with the rupture of a pulmonary artery aneurysm has been reported in the range of 50%-100% [6].

Effective management of this complex condition requires a tailored and multidisciplinary approach. Pulmonary artery aneurysms in BD stem from chronic inflammation and oxidative damage. Early detection and appropriate management are essential, given their substantial mortality risk. Male patients appear to be at greater risk, emphasizing the need for heightened vigilance during evaluation [7]. One key challenge in management is preventing organ damage. Management varies based on organ system involvement, severity, age, and gender. Colchicine serves as the cornerstone for mucocutaneous lesions, while other manifestations may necessitate agents like cyclosporine and anti-TNF agents [8]. Vascular involvement, attributed to endothelial inflammation and thrombosis, underscores the importance of reducing inflammation. Though the role of anticoagulation is debated, immunosuppressive agents such as corticosteroids, azathioprine, and cyclosporine A show promise in preventing thrombotic events [9]. Limited evidence exists for treating pulmonary artery aneurysms in Behçet's, but immunosuppressive approaches demonstrate efficacy. Intravenous corticosteroids and cyclophosphamide followed by oral corticosteroids with other immunosuppressive agents have resulted in improved survival rates [10].

While medical therapies have been reported to induce regression of aneurysms, recurrence remains common. Interventional techniques, such as coil embolization, are widely accepted for their ability to preserve vasculature, with a potential risk of rupture. Coil embolization is favored over thoracotomy due to the associated high mortality rate. Although the use of coils may lead to periprocedural rupture of the PAA, the actual risk remains theoretical and lacks documented evidence. The consideration of newer methods, such as stent-assisted coil embolization for larger aneurysms, is emerging. In the presented case, initial treatment with medical management failed to regress PAAs, necessitating endovascular coil embolization. Additional therapy involving methylprednisolone and cyclophosphamide, in conjunction with coil embolization, resulted in subsequent regression of the aneurysms.

The case study demonstrates successful management through pulmonary artery coil embolization, immunosuppressive therapy, and anti-inflammatory agents. Repeat imaging demonstrates the resolution of aneurysms without coiling, highlighting the benefit of comprehensive treatment. In conclusion, managing Behçet's disease with pulmonary artery involvement demands a patient-specific strategy. A coordinated effort, encompassing immunosuppression, interventional procedures, and anti-inflammatory agents, offers promising outcomes and underscores the importance of an integrated approach in achieving successful disease management.

## Patient consent

Written informed consent for the publication of “Pulmonary Artery Involvement in Behçet's Disease: A Challenging Case and Comprehensive Management Approach” was obtained from the patient. We have copy of the written consent on file.

## References

[1] Law N., Quencer K., Kaufman C., Iravani A., Hardman R., Smith T. (2022). Embolization of pulmonary artery aneurysms in a patient with Behçet's disease complicated by coil erosion into the airway. J Vasc Surg Cases, Innov Techniq.

[2] Greco A., De Virgilio A., Ralli M., Ciofalo A., Mancini P., Attanasio G. (2018). Behçet's disease: new insights into pathophysiology, clinical features and treatment options. Autoimmun Rev.

[3] Yazici H., Başaran G., Hamuryudan V., Hizli N., Yurdakul S., Mat C. (1996). The ten-year mortality in Behçet’s syndrome. Br J Rheumatol.

[4] Bilgin G., Sungur G., Kucukterzi V. (2013). Systemic and pulmonary screening of patients with Behçet's disease during periodic follow-up. Respir Med.

[5] Kage H., Goto Y., Amano Y., Makita K., Isago H., Kobayashi K. (2016). Development of pulmonary artery aneurysms due to Behçet's disease and resolution after treatment. Intern Med.

[6] Park HS., Chamarthy MR., Lamus D., Sachin (2018). Pulmonary artery aneurysms: diagnosis & endovascular therapy. Cardiovasc Diagnos Ther.

[7] Ucar-Comlekoglu D., Fox A., Sen HN. (2014). Gender differences in Behçet's disease associated uveitis. J Ophthalmol.

[8] Alibaz-Oner F., Direskeneli H. (2021). Advances in the treatment of Behcet's disease. Curr Rheumatol Rep.

[9] Seyahi E., Yurdakul S. (2011). Behçet's syndrome and thrombosis. Mediterranean J Hematol Infectious Dis.

[10] Hatemi G., Christensen R., Bang D., Bodaghi B., Ferhat Celik A., Fortune F. (2018). 2018 update of the EULAR recommendations for the management of Behçet's syndrome. A Rheumat Dis.

